# Disproportionate expansion of radical prostatectomy among older men in Japan in the robotic surgery era: a nationwide descriptive time series analysis

**DOI:** 10.1007/s11701-026-03817-4

**Published:** 2026-08-17

**Authors:** Tomohiko Hara, Suguru Oka, Kazushige Sakaguchi, Shinji Urakami

**Affiliations:** 1 Office of Research Administration, Center for Regulatory Science, Pharmaceuticals and Medical Devices Agency, Tokyo, Japan; 2 Department of Urology, Toranomon Hospital, Tokyo, Japan

**Keywords:** Robot-assisted surgery, Radical prostatectomy, Prostate cancer, Older patients, Claims data, Japan

## Abstract

Robot-assisted radical prostatectomy (RARP) has rapidly expanded and become the leading surgical approach for prostate cancer in Japan. However, it remains unclear whether the concurrent increase in radical prostatectomy reflects population aging, increased diagnostic activity, or shifts in treatment selection after diagnosis. We performed a nationwide descriptive time-series analysis using publicly available Japanese administrative claims, cancer registry data, population statistics, and hospital function reports. We identified prostate cancer surgeries, laboratory testing, and prostate biopsies through reimbursement codes and analyzed them as annual claim counts. Surgical procedures were categorized as open, laparoscopic, minimally invasive endoscopic, or RARP. Trends from 2016 to 2024 were evaluated, and age-specific metrics for men aged 75–79, 80–84, and ≥ 85 years were referenced to 2018. Prostate cancer surgeries rose from 21,988 in 2016 to 29,548 in 2024. RARP procedures increased from 13,415 to 27,911, comprising 94.5% of all surgeries in 2024. The share of surgeries in men aged ≥ 75 years grew from 12.3% to 30.5% and among men aged ≥ 80 years, it rose from 0.9% to 4.7%. For men aged 80–84 years, the 2024 surgery index reached 463.0, surpassing the indices for population size, incidence, mortality, prostate-specific antigen testing, and biopsy. Radical prostatectomy increased disproportionately among older men in Japan, particularly those aged 80–84 years, predominantly through the expansion of RARP. These population-level trends highlight the need for individualized patient selection based on frailty, life expectancy, tumor risk, and expected treatment benefit to mitigate potential overtreatment in older men.

**Supplementary Information:**

The online version contains supplementary material available at 10.1007/s11701-026-03817-4.

## Introduction

Prostate cancer ranks among the most common malignancies in men, with incidence rising sharply with age. Its importance in clinical and public health contexts is increasing, especially in aging populations like Japan. Since prostate cancer often develops slowly, the potential benefit of curative treatment largely depends on factors such as life expectancy, other mortality risks, tumor aggressiveness, and functional health. According to guidelines, definitive local treatment is usually recommended when a patient’s expected survival is approximately 5–10 years or more, based on tumor risk and symptoms [1, 2].

Over the last 20 years, prostate cancer treatment patterns in Japan have significantly evolved. Earlier large-scale studies indicated that primary androgen deprivation therapy was often chosen as the first treatment, even for cases without metastases [3]. Recent data indicate a move toward definitive local therapies, such as radical prostatectomy [4]. This transition has involved the widespread use of robot-assisted radical prostatectomy (RARP), which Japan’s national health insurance has covered since 2012, establishing it as a primary surgical method for localized prostate cancer.

The expanding use of curative-intent surgery in older patients is clinically challenging. For carefully selected patients with significant disease and a reasonable life expectancy, this approach might be considered a suitable treatment escalation. However, it also prompts concerns about overtreatment in those with limited life expectancy, frailty, or slow-progressing disease. According to the 2023 abridged life table, the life expectancy for Japanese men at ages 80 and 85 is roughly 8.98 and 6.29 years, respectively [5]. This underscores the importance of thorough, personalized evaluation before invasive curative procedures.

Although RARP can be performed safely in carefully selected older patients [6–10], chronological age alone does not determine treatment suitability. Older age and frailty are linked to poorer outcomes following prostatectomy, as well as higher mortality and complication rates in patients with prostate cancer [11, 12]. The main clinical challenge is to identify patients who are likely to gain meaningful benefit from surgery while avoiding unnecessary overtreatment. In Japan, population studies have raised concerns about potential overtreatment among very old men aged 80 or older [13]. In contrast, recent surveys indicate increasing consideration of active surveillance for selected patients with low- or intermediate-risk prostate cancer [14]. Given these competing considerations, it is still uncertain how curative-intent surgery is utilized for older and very old men with prostate cancer in everyday urological practice in Japan. There is limited nationwide data on age-specific trends in prostate cancer surgery [15] and related diagnostic processes, including prostate-specific antigen (PSA) testing and prostate biopsy.

To fill this gap, we conducted a nationwide descriptive time-series analysis using publicly available administrative and healthcare data in Japan. We evaluated the adoption of robot-assisted surgical systems via the Hospital Bed Function Report. We tracked age-specific annual trends in prostate cancer surgery, PSA testing, and prostate biopsies using the National Database of Health Insurance Claims and Specific Health Checkups of Japan Open Data (NDB Open Data). Additionally, we combined this information with prostate cancer incidence and mortality statistics from national cancer registries and demographic data to explore changes in surgical practices among older men, especially those aged 80 years or above.

## Methods

### Study design and data sources

This retrospective descriptive study utilized publicly available aggregate data to analyze national trends in population, prostate cancer incidence and mortality, diagnostic practices, prostate cancer surgeries, and the availability of treatment devices in Japan. The data sources comprised hospital function report data [16], NDB Open Data [17], National Cancer Registry statistics [18], and population and vital statistics [5]. These aggregate datasets did not permit record linkage at the patient or surgeon level and contained no patient-level information on PSA values, clinical stage, Gleason score or ISUP Grade Group, detailed biopsy findings, tumor burden, comorbidity, frailty, functional status, estimated life expectancy, perioperative outcomes, or long-term survival. Although RARP has been included in Japan’s national health insurance since 2012, it only became listed as a separate procedure category in the NDB Open Data starting in 2016. As a result, 2016 was chosen as the baseline year for analyses requiring identification of RARP at the procedure level. Data were available from 2016 to 2024 for hospital function report device counts, NDB claims, cancer mortality, and population figures, while data from the National Cancer Registry on cancer incidence and first-course treatment were available from 2016 to 2023; data for 2024 had not yet been published.

### NDB open data procedure definitions

Procedures and diagnostic tests were identified using Japanese reimbursement claim codes. Prostate cancer surgeries were categorized with the K843 code series: K843 for open surgery on malignant prostate tumors, K843-2 for laparoscopic procedures, K843-3 for minimally invasive endoscopic surgeries (MIES), and K843-4 for RARP. PSA testing was coded as 160,037,510 within the D009 tumor marker category. Prostate biopsies were identified using codes 160,098,110 and 160,231,810 in the D413 category, covering conventional prostate needle biopsies and MRI–ultrasound fusion-guided biopsies, respectively. For each year, inpatient and outpatient counts linked to these codes were combined. Since the NDB Open Data provides aggregated claim counts, these were analyzed as annual numbers of procedures or tests rather than unique patients. Repeated PSA tests, prostate biopsies, or procedure claims involving the same individual could not be identified, and the clinical indication for PSA testing and prostate biopsies could not be determined. Counts were summarized annually by 5-year male age groups and procedure categories as reported in the NDB Open Data.

### Handling masked and missing values

In the hospital function report data, blank cells and nonnumeric entries were considered missing. For each category, annual device availability was summarized by the total number of reported devices, the number of facilities reporting at least one device, and the count of records with missing device numbers.

Values shown as “–” in the NDB Open Data source tables indicated small-cell suppression, meaning counts of 9 or fewer were masked. Starting in 2022, zero counts were clearly differentiated from these suppressed “–” values. For age groups under 20 years, counts were consistently treated as 0 throughout the study, while suppressed “–” values in other age groups or procedure categories were imputed as 5 for the analysis. Since these suppressed cells represented small counts, this imputation was expected to have little impact on overall national trends. To assess the robustness of the findings to this assumption, sensitivity analyses were performed by alternatively imputing suppressed or missing NDB counts as 0 and 9.

### Outcomes and statistical analysis

Annual trends were summarized for treatment device availability, focusing on men’s population size, prostate cancer incidence and mortality, PSA testing, prostate biopsy counts, and prostate cancer surgery counts. The treatment devices analyzed included brachytherapy, robot-assisted surgical systems, and intensity-modulated radiation therapy (IMRT) devices. For each device type, we detailed both the total number of devices reported and the count of facilities with at least one device, separating overall and facility-specific availability. Prostate cancer surgeries were examined by age group and surgical method, classified as open, laparoscopic, MIES, or RARP. Age groups were redefined as ≤ 64, 65–69, 70–74, 75–79, 80–84, and ≥ 85 years.

Representative data on surgical and device trends were provided for 2016, 2020, and 2024. The age distribution of prostate cancer surgeries was analyzed by calculating the proportion performed in older age groups. Differences between 2016 and 2024 were evaluated using chi-square tests of proportions, with a two-sided *P*-value < 0.05 indicating statistical significance. These tests were used solely as supplementary descriptive comparisons of changes over time; interpretation focused primarily on absolute counts, percentages, and the magnitude of changes.

Additional analyses targeted men aged 75–79, 80–84, and ≥ 85 years. For each group, the male population, prostate cancer incidence and mortality, PSA testing, prostate biopsies, and surgeries were indexed to 2018, setting that year’s value at 100 due to consistent prostate biopsy reimbursement codes from then on. Annual data were reported from 2018 to 2024, except for prostate cancer incidence, for which 2023 was the latest available. To assess the relative growth of prostate cancer surgeries compared with other indicators, surgery-to-indicator index ratios were calculated by dividing the prostate cancer surgery index by each indicator’s index. Ratios above 1 indicated that surgery grew faster than the other indicators.

First-course treatment data from the National Cancer Registry were summarized descriptively based on reported combinations of surgery- and radiotherapy-related treatments. Since these treatment categories were not available by age group, they served only as supplementary contextual information and were not included in the age-specific indexed analyses.

### Software, editorial assistance, and data verification

Data extraction utilized Python 3.9.13 and the openpyxl package. Data were then aggregated and visualized using R 4.3.3 along with the dplyr, tidyr, ggplot2, scales, and patchwork packages. Tools like ChatGPT, Codex (OpenAI, San Francisco, CA, USA), and Positron (Posit Software, PBC, Boston, MA, USA) served solely as supplementary aids for code editing, data processing, and language refinement; they were not relied upon for autonomous data interpretation or statistical decisions. Professional English editing was performed by a commercial service. All datasets, extraction methods, code, results, and manuscript content underwent manual review by the authors to ensure accuracy and consistency.

## Results

### Treatment device availability

**Fig. 1:**
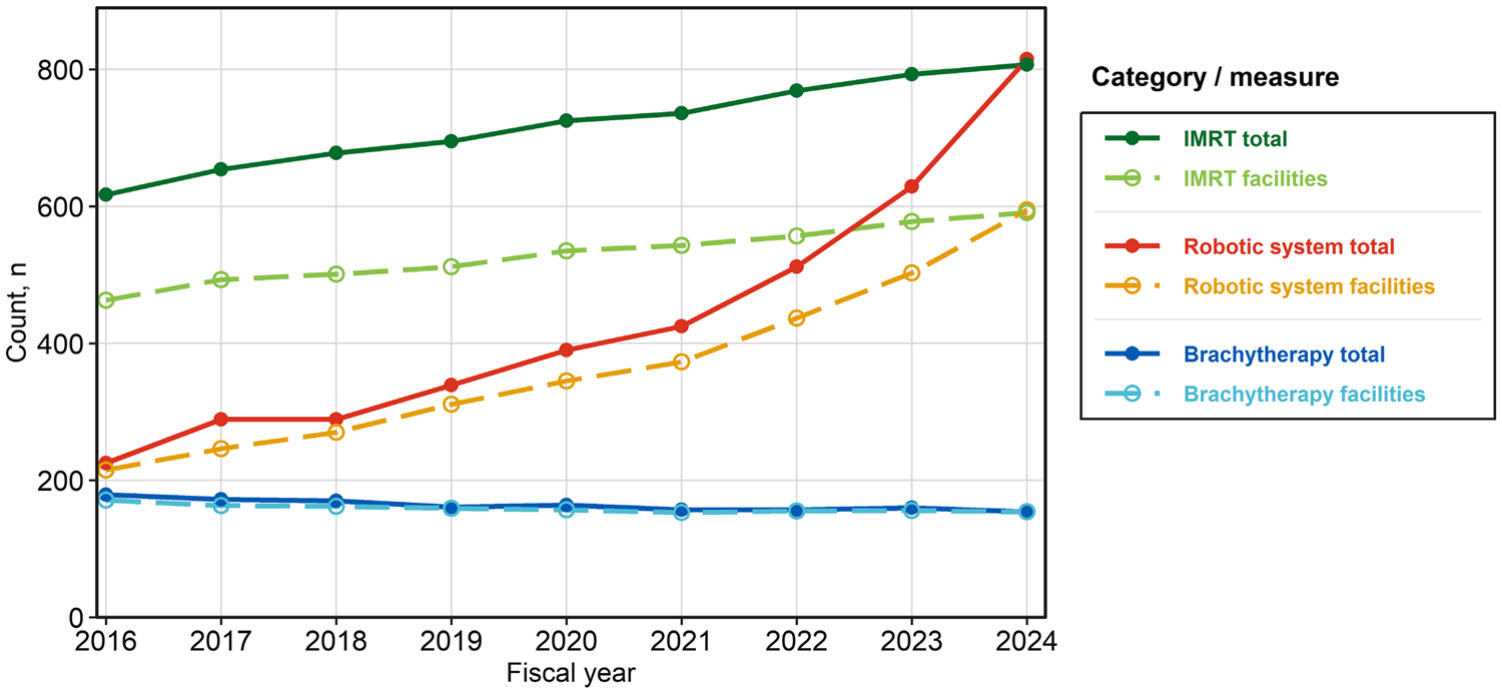
Trends in treatment-related infrastructure for prostate cancer in Japan, 2016–2024. It shows annual facility numbers and total treatment devices for intensity-modulated radiation therapy (IMRT), robot-assisted surgical systems, and brachytherapy. Solid lines represent the total device counts, while dashed lines show the number of facilities

Treatment device availability shifted significantly over the study period (Fig. 1). The largest growth was observed for robot-assisted surgical systems, with the total number rising from 225 in 2016 to 390 in 2020 and reaching 815 in 2024, a 262.2% increase from 2016 to 2024 (Table 1). Facilities with at least one robot-assisted surgical system grew from 215 to 345 and then to 595, representing a 176.7% increase. Meanwhile, the total number of IMRT devices increased modestly from 617 to 725 and 807 (+ 30.8%), and the number of facilities reporting at least one IMRT device rose from 463 to 535 and 591 (+ 27.6%). Conversely, the number of brachytherapy devices declined from 179 to 164 and 154 (− 14.0%), and the number of facilities reporting these devices decreased from 171 to 157 and 154 (− 9.9%).

**Table 1:** Comprehensive prostate cancer-related indicators according to year and age group

Indicator	2016			2020			2024		
Age group	< 75 y	75–79 y	≥ 80 y (80–84/ ≥ 85)	< 75 y	75–79 y	≥ 80 y (80–84/ ≥ 85)	< 75 y	75–79 y	≥ 80 y (80–84/ ≥ 85)
Prostate cancer surgery, total	19,290	2,500	198 (158/40)	18,740	3,948	493 (446/47)	20,529	7,646	1,373 (1,315/58)
Open surgery	4,480	610	45 (35/10)	1,527	366	62 (52/10)	442	217	39 (34/5)
Laparoscopic surgery	2,204	335	28 (18/10)	1,594	403	68 (58/10)	533	226	68 (63/5)
MIES	758	92	21 (11/10)	337	96	15 (5/10)	72	35	5 (5/0)
RARP	11,848	1,463	104 (94/10)	15,282	3,083	348 (331/17)	19,482	7,168	1,261 (1,213/48)
Male population, thousands	55,200	2,900	3,700 (2,100/1,600)	52,700	3,100	4,100 (2,200/1,900)	50,200	3,500	4,700 (2,600/2,200)
Prostate cancer incidence, thousands	48.5	18.8	22.4 (13.1/9.3)	43.1	19.9	24.6 (13.7/10.9)	*NA*	*NA*	*NA*
Prostate cancer mortality, thousands	2.6	1.9	7.3 (2.7/4.6)	2.4	2.0	8.3 (2.8/5.5)	2.2	2.2	9.2 (3.0/6.3)
PSA tests, thousands	1,872	609	739 (449/290)	1,744	622	796 (456/340)	1,828	756	958 (557/402)
Prostate biopsies, thousands	19.1	5.7	4.8 (3.4/1.4)	68.1	22.9	16.7 (11.9/4.8)	80.0	34.7	25.7 (18.9/6.8)
Robot-assisted surgical systems	225			390			815		
IMRT devices	617			725			807		
Brachytherapy devices	179			164			154		
Facilities with robot-assisted surgical systems	215			345			595		
Facilities with IMRT devices	463			535			591		
Facilities with brachytherapy devices	171			157			154		

Population values were rounded to the nearest 100,000, PSA testing counts to the nearest 1,000, and incidence, mortality, and biopsy counts to the nearest 100. For the ≥ 80-year group, values in parentheses indicate data for men aged 80–84 years and ≥ 85 years, respectively. NA denotes not available; IMRT, intensity-modulated radiation therapy; MIES, minimally invasive endoscopic surgery; NDB, National Database of Health Insurance Claims and Specific Health Checkups of Japan; PSA, prostate-specific antigen; RARP, robot-assisted radical prostatectomy

### Prostate cancer surgery by age group

**Fig. 2:**
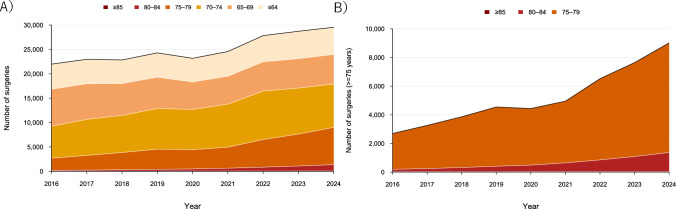
Age-specific trends in prostate cancer surgery in Japan, 2016–2024. (**A**) Yearly prostate cancer surgeries segmented by age groups. (**B**) Yearly prostate cancer surgeries for men aged 75 and above, divided into 75–79, 80–84, and 85 years or older

The total number of prostate cancer surgeries grew from 21,988 in 2016 to 23,181 in 2020 and 29,548 in 2024, marking an overall rise of approximately 34% over these years (Fig. 2A and Table 1). This increase was particularly significant among older age groups (Fig. 2B). For men aged 75–79 years, surgeries rose from 2,500 in 2016 to 3,948 in 2020 and reached 7,646 in 2024, a 3.1-fold rise. For those aged 80–84 years, the numbers increased from 158 to 446 and 1,315, representing an 8.3-fold increase. Men aged 85 or older saw a smaller increase, from 40 to 47 and 58 surgeries, equating to a 1.5-fold rise.

The percentage of surgeries in men aged 75 years or older rose from 12.3% in 2016 to 19.2% in 2020 and reached 30.5% in 2024; this increase from 2016 to 2024 was statistically significant (*P* < 0.001). For men aged 80 years or older, the proportions were 0.9%, 2.1%, and 4.7%, respectively. In the 80–84 age group, the proportions increased from 0.7%, to 1.9%, then to 4.5%, with a significant rise from 2016 to 2024 (*P* < 0.001). Conversely, men aged 85 years or older constituted only 0.18%, 0.20%, and 0.20%, respectively, showing no significant change during this period (*P* = 0.70). Sensitivity analyses using alternative imputations of 0 and 9 confirmed that the principal age-specific findings were robust and were not materially affected by the handling of suppressed or missing NDB counts (Supplementary Tables S1 and S2).

### Surgical approach

**Fig. 3:**
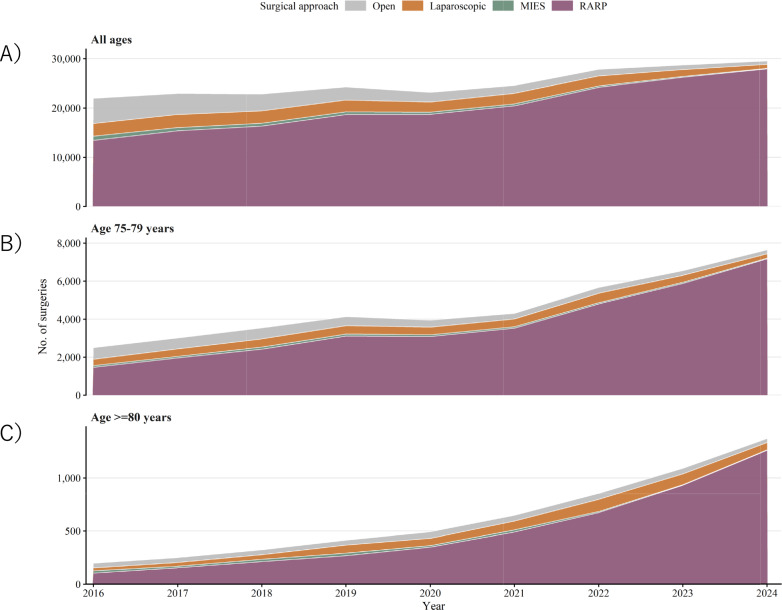
Trends in surgical methods for prostate cancer across different age groups, 2016–2024. The stacked area charts display yearly counts of prostate cancer surgeries by approach for (**A**) all ages, (**B**) men aged 75–79, and (**C**) men aged 80 and above. Gray represents open surgery, orange represents laparoscopic surgery, green represents minimally invasive endoscopic surgery (MIES), and purple represents robot-assisted radical prostatectomy (RARP)

RARP experienced rapid growth and became the leading surgical method throughout the study period (Table 1). The total number of RARP procedures rose from 13,415 in 2016 to 18,713 in 2020 and 27,911 in 2024, making up 61.0%, 80.7%, and 94.5% of all prostate cancer surgeries, respectively (Fig. 3A). During the same time, open surgeries declined from 5,135 to 1,955 and 698; laparoscopic surgeries decreased from 2,567 to 2,065 and 827; and MIES procedures dropped from 871 to 448 and 112.

The predominance of RARP was also evident in older men. For those aged 75–79 years, RARP procedures rose from 1,463 in 2016 to 3,083 in 2020, and reached 7,168 in 2024, accounting for 58.5%, 78.1%, and 93.7% of surgeries in this group, respectively (Fig. 3B). In men aged 80 and above, RARP increased from 104 to 348 and then to 1,261 cases, representing 52.5%, 70.6%, and 91.8% of surgeries, respectively (Fig. 3C). By 2024, open, laparoscopic, and MIES surgeries accounted for only small shares of overall procedures in both older age groups.

### Indexed trends among older men

**Fig. 4:**
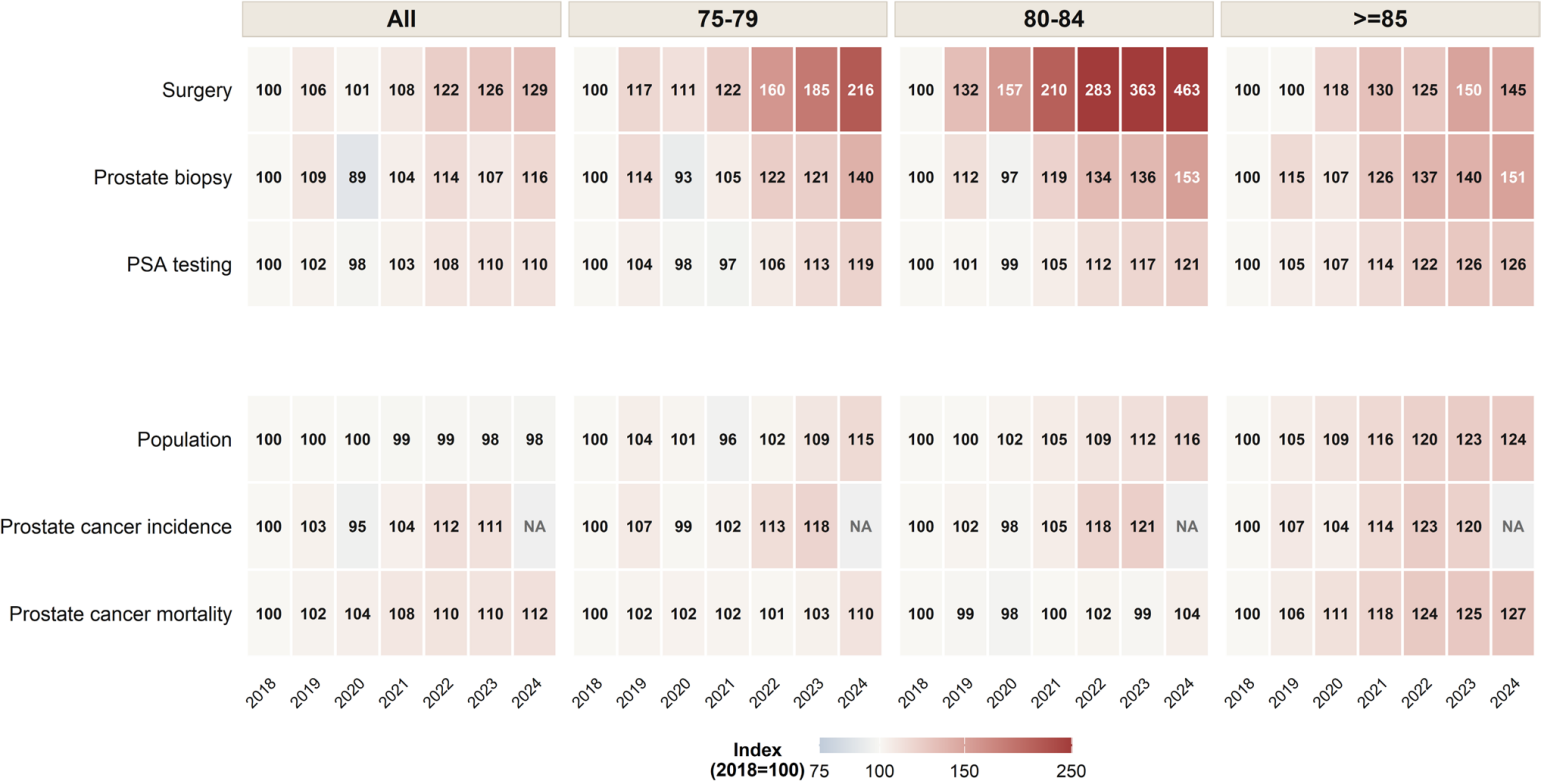
Indexed trends in prostate cancer-related indicators among older men, 2018–2024. Heatmaps show age-specific changes in population size, prostate cancer incidence, mortality, prostate-specific antigen (PSA) testing counts, prostate biopsy counts, and prostate cancer surgery counts for all men combined and for men aged 75–79, 80–84, and 85 years or older. All indicators were indexed to 2018, with the 2018 value set to 100. Cell values indicate the corresponding index values. Prostate cancer incidence data were available only through 2023; therefore, incidence values for 2024 are missing

Figure 4 shows indexed trends in the male population, including prostate cancer incidence, mortality, PSA testing, prostate biopsies, and surgeries. These are presented for all men and specifically for those aged 75–79, 80–84, and 85 years or older, with 2018 as the baseline at 100. Incidence data extend through 2023, while the other indicators are available up to 2024.

For men aged 75–79 years, the latest index values were 115.0 for the general male population, 118.0 for incidence, 109.9 for mortality, 118.8 for PSA testing counts, 140.5 for biopsy counts, and 215.9 for surgery counts. In men aged 80–84 years, the figures were 116.4, 120.9, 104.4, 120.7, 153.5, and 463.0, respectively. For men aged 85 years or older, the values were 124.0, 120.0, 126.7, 126.1, 151.1, and 145.0, respectively.

Surgery counts rose more quickly than other indicators in men aged 75–79 and 80–84 years, but not in those aged 85 or older. The surgery-to-biopsy index ratio was highest among men aged 80–84 years at 3.02. In this age group, surgery increased from 284 procedures in 2018 to 1,315 in 2024 (index, 463.0), whereas prostate biopsies increased from 12,306 to 18,890 (index, 153.5). Among men aged 75–79 years, surgery increased from 3,542 to 7,646 (index, 215.9), and biopsies from 24,675 to 34,668 (index, 140.5), yielding a surgery-to-biopsy index ratio of 1.54. Among men aged ≥ 85 years, surgery increased from 40 to 58 (index, 145.0), and biopsies from approximately 4,490 to 6,785 (index, 151.1), yielding a ratio of 0.96.

## Discussion

This nationwide analysis presents three key findings. Firstly, prostate cancer surgery in Japan has shifted significantly toward RARP, which now makes up the majority of procedures. Second, the rise in surgeries was especially notable among older men, particularly those aged 75–79 and 80–84 years. Third, in these two age groups, surgical numbers grew faster than PSA tests, prostate biopsies, prostate cancer incidence, mortality, and population growth, a pattern not seen among men aged 85 or older. These results suggest that RARP adoption may have influenced the boundary between what is technically feasible and what is clinically appropriate for men aged 75–79 and 80–84 years. Conversely, the low frequency of surgery among men aged 85 or older suggests that factors such as frailty, functional reserve, life expectancy, and expected benefits likely still limit surgical expansion at these ages. However, patient data are required to verify this.

Although prostate biopsy counts also increased among men aged 75–79 and 80–84 years during the study period, surgery counts rose even more, suggesting that the observed trend may reflect not only increased diagnostic activity but also a shift in treatment selection after diagnosis. Careful patient selection may have contributed to this pattern, but the magnitude of the increase in surgery relative to diagnostic indicators suggests it may not fully explain the trend. On the supply side, the rapid adoption of robot-assisted surgical systems may have made curative-intent surgery more readily available to older patients. As these systems became more widely available and surgical teams gained experience, surgery may have been offered to a broader range of patients who were previously less likely to undergo it, a pattern compatible with supply-sensitive diffusion [19] and indication creep [20]. At the system level, Japan’s universal health insurance coverage, widespread medical infrastructure, and relatively equitable access to specialized care may have facilitated access to definitive treatment after diagnosis. At the patient level, preserved functional status, preferences for definitive treatment, family expectations, and favorable perceptions of RARP as a less invasive surgical approach may further shape treatment decisions among older men [21]. Similar shifts in surgical practice have been reported internationally, with increasing use of prostatectomy among older patients in population-based data from Europe [22, 23].

In the overall population, RARP progressively replaced open, laparoscopic, and minimally invasive endoscopic approaches during the study period. Recent randomized and meta-analytic evidence indicates that, compared with open radical prostatectomy, RARP is associated with reduced blood loss, lower transfusion requirements, shorter hospitalization, and fewer overall postoperative complications, while maintaining oncologic efficacy [24–28]. These advantages may have lowered the practical threshold for considering radical prostatectomy in carefully selected older men who might previously have been regarded as poor candidates for conventional open surgery because of concerns regarding operative invasiveness and postoperative recovery. Supporting this possibility, recent multicenter data suggest that RARP can be performed safely and feasibly in carefully selected men aged ≥ 80 years, including patients with high-risk or locally advanced disease [8]. However, the present data cannot determine whether greater availability of RARP directly influenced treatment decisions or whether the observed increase reflected changes in patient selection, tumor characteristics, or broader management strategies.

For older patients, technical feasibility and perioperative tolerability should not be assumed to be equivalent to clinical appropriateness, meaningful patient benefits, or health-system value—especially given the high costs of robotic surgery [29]. Among men aged ≥ 85 years, the increase in surgery was broadly proportional to the increase in biopsy activity, whereas surgery increased far more markedly than biopsy among men aged 80–84 years. This contrast suggests that the findings in men aged 80–84 years cannot be explained solely by increased diagnostic activity and may also reflect an expansion of surgical eligibility in the robotic surgery era. In a nationally representative survey of frailty among community-dwelling older Japanese adults, the proportion classified as robust declined from 33.1% at ages 80–84 years to 17.1% at ages ≥ 85 years [30]. This may indicate that the lower invasiveness of RARP does not overcome the fundamental limits of curative surgery in very old patients with severe comorbidity, frailty, limited functional reserve, or insufficient life expectancy. These findings emphasize the importance of individualized surgical decision-making rather than reliance on chronological age alone. Consistent with current guidelines, older patients considered for RARP should undergo individualized assessment of life expectancy, comorbidity, frailty, functional status, and cognition using validated tools such as the Geriatric 8, Mini-Cog, Charlson Comorbidity Index, and Clinical Frailty Scale [1]. From a healthcare system perspective, they highlight the need to assess whether increased use of RARP among older and very old men offers meaningful patient benefits and value within a publicly funded healthcare system.

Several limitations should be noted. First, this study used aggregated NDB Open Data, which precluded analysis at the individual patient, surgeon, or hospital level. Because these aggregate datasets lacked patient-level clinical, oncological, and health-status information, we could not determine whether the increase in surgery among older men reflected appropriate treatment of fit patients with clinically significant localized disease or potential overtreatment of men unlikely to benefit from radical therapy. In addition, PSA testing, prostate biopsies, and surgeries were recorded as claim counts rather than unique patients, and repeated diagnostic claims could not be distinguished at the individual level. We were also unable to assess whether the diffusion of RARP improved perioperative or long-term oncological outcomes. Second, although RARP has been covered by Japan’s national health insurance since 2012, analyses were limited to 2018 onward because of consistent prostate biopsy reimbursement codes from that year, which prevented evaluation of early RARP adoption alongside similar diagnostic indicators. Third, this study did not capture the full spectrum of prostate cancer management, including IMRT, brachytherapy, active surveillance, watchful waiting, and androgen deprivation therapy. Therefore, we could not determine whether the observed increase in surgery represented a true expansion of treatment or a shift from other management strategies. Although supplementary National Cancer Registry data indicated broader shifts in definitive local treatment patterns (Supplementary Figure S1), these categories were not age specific. They thus did not directly explain the disproportionate rise in surgery among men aged 75–84. Fourth, the surgery-to-biopsy comparison was based on aggregate claim counts rather than unique patients and should therefore be interpreted only as an indirect comparison of surgical and diagnostic activity, not as the proportion of biopsied men who subsequently underwent surgery. Finally, as an ecological time-series analysis, the parallel increase in robot-assisted surgical systems and RARP use does not establish causality. The study could not determine whether the trend represented appropriate treatment intensification or potential overtreatment.

In summary, the rapid diffusion of RARP in Japan occurred alongside a disproportionate increase in radical prostatectomy among older men, especially those aged 80–84 years. This pattern suggests that the diffusion of RARP may have expanded surgical eligibility among older patients, raising questions about the balance between technical feasibility and clinical appropriateness. The subsequent challenge is to assess whether this broader surgical eligibility results in meaningful benefits for patients, such as improved functional and oncologic outcomes, and whether it offers sufficient value within a publicly funded healthcare system.

## Supplementary Information

Below is the link to the electronic supplementary material.

Supplementary file 1. Aggregated first-course treatment trends for prostate cancer in Japan, 2016–2023. Data were obtained from Japan’s National Cancer Registry. Red lines indicate surgery/endoscopy-related treatment and blue lines indicate radiotherapy-related treatment. Panel A shows the annual proportion of registered patients receiving each treatment category, and Panel B shows the corresponding estimated number of patients. National Cancer Registry data were available only through 2023; corresponding data for 2024 were not available

Supplementary file 2.

## Data Availability

All data used in this study are publicly available from Japanese government sources and national statistics, including the National Database of Health Insurance Claims and Specific Health Checkups of Japan Open Data, cancer statistics, population data, and hospital function reports. Independent researchers can reproduce the study dataset using these publicly accessible sources and the procedure definitions described in the Methods. The processed analytic dataset and analysis code are available from the corresponding author upon reasonable request.

## Abbreviations

IMRT: Intensity-modulated radiation therapy
MIES: Minimally invasive endoscopic surgery
NDB: National database of health insurance claims and specific health checkups of Japan
PSA: Prostate-specific antigen
RARP: Robot-assisted radical prostatectomy

## Declarations

### Disclaimer

The opinions in this article belong to the authors and may not represent the official stance or policies of the Pharmaceuticals and Medical Devices Agency.
