# Supplementary material for: Disproportionate expansion of radical prostatectomy among older men in Japan in the robotic surgery era: a nationwide descriptive time series analysis
Source: J Robot Surg. 2026 Aug 17;20(1):844. doi: 10.1007/s11701-026-03817-4 (PMC13481760; doi:10.1007/s11701-026-03817-4)
Supplement: Supplementary file 1 [file 11701_2026_3817_MOESM2_ESM.docx]

**Supplemental Table S1. Comprehensive Prostate Cancer-Related Indicators According to Year and Age Group (Sensitivity analysis: suppressed or missing NDB counts were imputed as 0)**

| **Indicator** | **2016** | | | | **2020** | | | | **2024** | | | |
| --- | --- | --- | --- | --- | --- | --- | --- | --- | --- | --- | --- | --- |
|  | **Under 75 yr** | **75-79 yr** | **80-84 yr** | **85 yr or older** | **Under 75 yr** | **75-79 yr** | **80-84 yr** | **85 yr or older** | **Under 75 yr** | **75-79 yr** | **80-84 yr** | **85 yr or older** |
| Prostate cancer surgery, total (K843 series) | 19,180 | 2,500 | 158 | 0 | 18,620 | 3,948 | 441 | 12 | 20,499 | 7,646 | 1,310 | 43 |
| Open surgery | 4,455 | 610 | 35 | 0 | 1,497 | 366 | 52 | 0 | 432 | 217 | 34 | 0 |
| Laparoscopic surgery | 2,174 | 335 | 18 | 0 | 1,564 | 403 | 58 | 0 | 528 | 226 | 63 | 0 |
| Minimally invasive endoscopic surgery (MIES) | 728 | 92 | 11 | 0 | 302 | 96 | 0 | 0 | 62 | 35 | 0 | 0 |
| Robot-assisted surgery | 11,823 | 1,463 | 94 | 0 | 15,257 | 3,083 | 331 | 12 | 19,477 | 7,168 | 1,213 | 43 |
| Male population, thousands | 55,200 | 2,900 | 2,100 | 1,600 | 52,700 | 3,100 | 2,200 | 1,900 | 50,200 | 3,500 | 2,600 | 2,200 |
| Prostate cancer incidence | 48,522 | 18,815 | 13,122 | 9,258 | 43,149 | 19,930 | 13,735 | 10,942 | *NA* | *NA* | *NA* | *NA* |
| Prostate cancer mortality | 2,582 | 1,927 | 2,696 | 4,605 | 2,408 | 2,054 | 2,767 | 5,530 | 2,206 | 2,209 | 2,958 | 6,297 |
| PSA testing | 1,872,019 | 609,248 | 448,576 | 289,971 | 1,743,639 | 622,367 | 456,011 | 339,967 | 1,828,174 | 756,195 | 556,735 | 401,668 |
| Prostate biopsy | 19,080 | 5,729 | 3,449 | 1,390 | 68,147 | 22,915 | 11,912 | 4,822 | 79,940 | 34,668 | 18,890 | 6,785 |

*NA denotes not available; MIES, minimally invasive endoscopic surgery; NDB, National Database of Health Insurance Claims and Specific Health Checkups of Japan; PSA, prostate-specific antigen.*

**Supplemental Table S2. Comprehensive Prostate Cancer-Related Indicators According to Year and Age Group (Sensitivity analysis: suppressed or missing NDB counts were imputed as 9)**

| **Indicator** | **2016** | | | | **2020** | | | | **2024** | | | |
| --- | --- | --- | --- | --- | --- | --- | --- | --- | --- | --- | --- | --- |
|  | **Under 75 yr** | **75-79 yr** | **80-84 yr** | **85 yr or older** | **Under 75 yr** | **75-79 yr** | **80-84 yr** | **85 yr or older** | **Under 75 yr** | **75-79 yr** | **80-84 yr** | **85 yr or older** |
| Prostate cancer surgery, total (K843 series) | 19,378 | 2,500 | 158 | 72 | 18,836 | 3,948 | 450 | 75 | 20,553 | 7,646 | 1,319 | 70 |
| Open surgery | 4,500 | 610 | 35 | 18 | 1,551 | 366 | 52 | 18 | 450 | 217 | 34 | 9 |
| Laparoscopic surgery | 2,228 | 335 | 18 | 18 | 1,618 | 403 | 58 | 18 | 537 | 226 | 63 | 9 |
| Minimally invasive endoscopic surgery (MIES) | 782 | 92 | 11 | 18 | 365 | 96 | 9 | 18 | 80 | 35 | 9 | 0 |
| Robot-assisted surgery | 11,868 | 1,463 | 94 | 18 | 15,302 | 3,083 | 331 | 21 | 19,486 | 7,168 | 1,213 | 52 |
| Male population, thousands | 55,200 | 2,900 | 2,100 | 1,600 | 52,700 | 3,100 | 2,200 | 1,900 | 50,200 | 3,500 | 2,600 | 2,200 |
| Prostate cancer incidence | 48,522 | 18,815 | 13,122 | 9,258 | 43,149 | 19,930 | 13,735 | 10,942 | *NA* | *NA* | *NA* | *NA* |
| Prostate cancer mortality | 2,582 | 1,927 | 2,696 | 4,605 | 2,408 | 2,054 | 2,767 | 5,530 | 2,206 | 2,209 | 2,958 | 6,297 |
| PSA testing | 1,872,019 | 609,248 | 448,576 | 289,971 | 1,743,639 | 622,367 | 456,011 | 339,967 | 1,828,174 | 756,195 | 556,735 | 401,668 |
| Prostate biopsy | 19,143 | 5,729 | 3,449 | 1,390 | 68,201 | 22,915 | 11,912 | 4,822 | 80,021 | 34,668 | 18,890 | 6,794 |

*NA denotes not available; MIES, minimally invasive endoscopic surgery; NDB, National Database of Health Insurance Claims and Specific Health Checkups of Japan; PSA, prostate-specific antigen.*
